# Factors associated with educational aspirations among adolescents: cues to counteract socioeconomic differences?

**DOI:** 10.1186/1471-2458-10-154

**Published:** 2010-03-24

**Authors:** Andrea Madarasova Geckova, Peter Tavel, Jitse P van Dijk, Thomas Abel, Sijmen A Reijneveld

**Affiliations:** 1 Kosice Institute for Society and Health, Medical Faculty, PJ Šafárik University, Tr SNP 1, 04101 Kosice, Slovak Republic; 2 Department of Psychology, Faculty of Arts, University of Olomouc, Vodarni 6, 771 80 Olomouc, Czech Republic; 3 Department Social Medicine, University Medical Center Groningen, University of Groningen, Ant Deusinglaan 1, 9713 AV Groningen, The Netherlands; 4 Institute of Social and Preventive Medicine, Medical Faculty, University of Bern, Bern, Switzerland

## Abstract

**Background:**

Our study aims to follow this effort and to explore the association between health, socioeconomic background, school-related factors, social support and adolescents' sense of coherence and educational aspirations among adolescents from different educational tracks and to contribute to the existing body of knowledge on the role of educational aspirations in the social reproduction of health inequalities. We expect that socioeconomic background will contribute to the development of educational aspirations, but this association will be modified by available social and individual resources, which may be particularly favourable for the group of adolescents who are on lower educational tracks, since for them such resources may lead to gaining a higher educational level.

**Methods:**

We collected data on the socioeconomic background (mother's and father's education and employment status, doubts about affordability of future study), school-related factors (school atmosphere, school conditions, attitudes towards school), perceived social support, sense of coherence (manageability, comprehensibility, meaningfulness) and the self-rated health of a national sample of Slovak adolescents (n = 1992, 53.5% females, mean age 16.9 years). We assessed the association of these factors with educational aspirations, overall and by educational tracks (grammar schools, specialised secondary schools, vocational schools).

**Results:**

We found statistically significant associations with educational aspirations for the factors parental educational level, father's unemployment, doubts about the affordability of future study, school atmosphere, attitude towards school, social support from the father and a sense of coherence. Social support from the mother and friends was not associated with educational aspiration, nor was self-rated health. Besides affinity towards school, the determinants of educational aspirations differed among adolescents on different educational tracks. Educational aspirations of grammar school students were associated with father's education, while the aspirations of their peers on lower educational tracks had a stronger association with mother's education and perceived social support from father and friends. Moreover, a sense of coherence contributes to the reporting of educational aspiration by students on different educational tracks.

**Conclusions:**

Characteristics of the school environment, the family and the individual adolescent are all associated with the level of educational aspiration, but in a different way for different educational tracks. Interventions aimed at reducing socioeconomic inequalities in health via the educational system should, therefore, take this variation and the rather pivotal role of the father into account.

## Background

Recent findings from a large international study on tackling inequalities in health (EUROTHINE) indicate that socioeconomic inequalities in health are present throughout Europe and that there are enormous opportunities for reducing them in many countries [1,2]. Reducing such inequalities in health is undoubtedly an important target of public health policy. It has particular relevance for youth, since young people still have great potential for avoiding the undesirable, cumulating consequences of social disadvantage.

Socioeconomic attainment is strongly linked to educational attainment [3,4]. Trajectory through an educational system might be one of the key issues to understanding the pathways by which social and economic background lead to future inequalities in health. Institutionalized cultural capital, e.g. formal education, plays a crucial role, but appears to be strongly dependent on the availability of sufficient incorporated cultural capital (an affinity for higher education, the motivation to invest in educational degrees), which is provided by parents via transmitting the attitudes and knowledge needed to succeed in the existing educational system [5,6]. Educational aspirations or educational expectations might be a good proxy measure of a more hidden element of cultural capital. Therefore understanding the role of educational aspirations in the social reproduction of health inequalities might be an important clue for strategies aiming to reduce inequalities in health. We might expect to find differences in educational aspirations between adolescents having different socioeconomic origins [7], but we need to know more about the pathways involved. Evidence on the association between socioeconomic background and educational aspirations of offspring are somewhat conflicting, indicating direct as well as indirect pathways [8-11]. The strong influence of socioeconomic background (parents' education, occupation, family income) on educational expectations was reported by Trusty [8] in his study of US adolescents. While Marjoribanks and Mboya [9] reported unmediated relations of family SEP with the adolescents' learning and performance-goal orientation, Garg et al[10] confirmed only an indirect effect of socioeconomic background on educational aspirations mediated via personal factors (academic achievement, parental educational expectation, perception of courses, extracurricular readings, importance of school and homework) and through parental involvement. Based on the cohort study by Mateju and Smith [11], we might expect a change from direct to indirect effects of social origin on educational aspirations in the Central European context because of the huge societal changes during the past 20 years that greatly expanded access to education and increased the value of higher educational attainment in these countries.

Adverse childhood conditions might affect educational chances, job opportunities and life chances in general, but this process might be ameliorated via available social sources (supportive family and school environment) and individual sources (sense of coherence) [12]. A supportive family [8,13-15] and school environment [7,10,16,17] contribute to the development of educational aspirations. However, while parental support seems to be more influential within lower and middle SEP adolescents, school attitudes had a stronger effect on educational expectations for upper SEP adolescents in a study of Australian adolescents [7,17]. A sense of coherence correlated significantly with school performance, but not with the selection of educational tracks in a study of Swedish adolescents [18].

Based on the theory of health selection [19,20], health disadvantage represented by frequent health complaints, worse well-being as well as more frequent school absence, might distort the development of educational aspirations, lead to underachievement and finally to downward mobility.

From the study of Mau & Bikos [7] we know that the school programme (academic track) among US adolescents was the strongest predictor of educational aspirations, but it is not yet known how educational aspiration is determined within particular educational tracks. As with having a better education, higher educational aspiration is important across all educational tracks and it might be important to know how those students allocated to vocational tracks differ in setting up their educational aspirations in comparison to those allocated to more academic tracks.

Our study aims to follow this effort and to explore the association between health, socioeconomic background, school-related factors, social support and adolescents' sense of coherence and educational aspirations among adolescents from different educational tracks and to contribute to the existing body of knowledge on the role of educational aspirations in the social reproduction of health inequalities.

## Methods

### Sample

The sample consisted of 1992 Slovak adolescents (53.5% females) from 24 secondary schools located in Kosice (240,000 inhabitants), a city in Eastern Slovakia. The schools and the classes in schools were chosen randomly, stratified by the five educational levels of the regular Slovak school system. Data were collected in the winter of 2002. Parents were informed prior to the study via the school administration in a regular meeting of parents with the staff of the school and could opt out if they disagreed with their child's participation. Children were informed prior to the study; participation in the study was fully voluntary and anonymous with no explicit incentives provided for participation. Data were collected in classrooms, in the absence of teachers. Students of first and third grades were approached. The mean age of respondents was 16.9 (SD = 1.1, 14-23 years). A response rate of 97.5% was achieved. Non-response was mainly due to illness.

The study was done according to the ethical requirements formulated by Agreement on Human Rights and Biomedicine (40/2000 Z.z.). The Science and Technology Assistance Agency in April 2002 approved in its decision on APVT-20-003602 also the ethical aspects of the study.

### Measures

Self-rated health was measured with one item of the SF-36 [21]. Respondents were asked to assess their health as (1) excellent, (2) very good, (3) good, (4) fairly good or (5) bad. The last three responses were merged into one category. This measure is widely used in health studies as an indicator of general health status, because it is generally accepted as a good predictor of mortality and morbidity [22,22].

To measure socioeconomic status, four indices were used: highest completed education of the father and mother and employment status of the father and mother. Three levels of education were distinguished: university education, secondary school education (with a leaving certificate), and vocational education or lower (without a leaving certificate). Respondents were asked to indicate whether their father and mother were employed or unemployed. Moreover, respondents were asked if they tended to have doubts about the affordability of further study. Respondents could choose one of five responses: (1) never, (2) rarely, (3) sometimes, (4) often and (5) very often. The last two responses were merged into one category.

Perceived social support from father, mother and friends was measured using 18 items derived from the Measure of Perceived Social Support [23]. These items focus on the following aspects: closeness with the respondent, availability for chatting with the respondent, expressing worth to the respondent, feeling relaxed when being together, being available when needed and confidence in the respondent. Each item has a four-point response scale (1-4) with the sum score ranging from 6 to 24 separately for father's support, mother's support and friends' support. A higher score indicates lower social support. The internal reliability of this questionnaire is highly satisfactory. Cronbach's alpha coefficients are 0.92 for father's support, 0.87 for mother's support and 0.86 for friends' support; mean inter-item correlations were 0.54, 0.65 and 0.50, respectively.

Among school-related variables, we measured school atmosphere, study conditions at school and attitudes towards school. Respondents were asked to choose one of three responses describing school atmosphere as (1) relaxed, friendly, feeling well and safe at school, (2) moderately tensed, irritated but bearable, and finally (3) tensed, irritated, not feeling safe at school, unbearable. The last two responses were merged into one category. Under study conditions we included classroom conditions, quantity and quality of educational aids and quality of the educational process. Respondents could choose one of three responses: (1) excellent conditions - appropriate classrooms, enough aids of satisfactory quality, good team of teachers, (2) sufficient conditions - something is missing, and (3) insufficient conditions - most of the named attributes are missing. Attitudes towards school were tested with one item. Respondents were asked if they usually liked school and could choose one of four responses: (1) I like it a lot; (2) I like it moderately; (3) I do not like it very much; and (4) I do not like it at all. The last two responses were merged into one category.

A sense of coherence was measured using the shortened, 13-item version of the Antonovsky scale [24]. A 5-point Likert scale (1-5) was used to answer items. The sum score varies from 4 to 20 in the dimensions of manageability and comprehensibility and from 5 to 25 in the dimension of meaningfulness. A higher score indicates a higher sense of coherence. Cronbach's alphas for these scales were 0.43 (manageability), 0.57 (comprehensibility) and 0.43 (meaningfulness), while for the whole scale it was 0.72. Mean inter-item correlations were 0.16, 0.21, 0.16 and 0.16, respectively. According to the guideline of Briggs & Cheek [25], the MIIC should range above .20, but not be less than 0.15 [26,27]. Additional analyses showed that only with the Comprehensibility scale would the internal consistency be improved by omitting two items (Cronbach alfa 0.57?0.65 MIIC 0.21?0.39). However, this small increase in an already rather good consistency would be at the expense of decreasing the comparability of our findings with others that were based on the original version of the scale. For this reason, we decided to use the original version.

Educational aspiration was measured with one item. Respondents were asked what they thought they would do in the future. They could choose one of five responses: (1) I will study in future; (2) I will be unemployed; (3) I will work, or I will go abroad; (4) I will start my own business; (5) I do not know. The item was dichotomised into those who planned to study in the future (i.e. 'yes') and the rest of the respondents (i.e. 'no').

Three educational tracks were distinguished. After leaving elementary school (9 years attendance), Slovak adolescents age 15 ± enter one of the three types of secondary schools: (1) a four-year grammar school providing general education and preparation for university study; (2) a four-year specialised secondary school usually providing technical education, after which it is also possible to study at university (However, this is a less academic education than grammar school.); and (3) a four-year vocational school providing education for manual occupations, after which it is also possible to study at university and a three or two-year vocational school providing only basic education for manual occupations. Our sample was made up of 24.9% grammar school students, 37.3% specialised secondary school students and 37.8% vocational school students.

### Analyses

The effects of self-rated health, socioeconomic background, school-related factors, social support as well as adolescents' sense of coherence on educational aspirations were tested using logistic regression (forward stepwise), adjusting for age and gender. As a second step the same model was also tested in three different educational tracks: among grammar school students, among specialised school students and among vocational school students.

## Results

The background characteristics, mean scores and standard deviations of the studied variables are presented in Table 1. Educational aspirations differed with statistical significance among students from different educational tracks (chisquare test, p < .001). While 86.0% of grammar schools students reported they wanted to study in the future, only 56.1% of specialised secondary school students and even fewer (28.1%) apprentices reported this for themselves.

**Table 1:** Selected factors of educational aspirations among adolescents: descriptive data

		All respondents	Grammar schools	Specialised sec. schools	Vocational schools
Age *		16.86 (1.11)	16.77 (1.12)	16.74 (1.06)	17.02 (1.13)

Gender	male	927 (46.5)	207 (41.7)	316 (42.5)	404 (53.7)
	female	1065 (53.5)	289 (58.3)	428 (57.5)	348 (46.3)

Self-rated health	excellent	507 (25.5)	112 (22.6)	197 (26.6)	198 (26.4)
	very good	819 (48.8)	246 (49.6)	324 (43.7)	249 (33.2)
	good, fairly good, bad	660 (30.6)	138 (27.8)	220 (29.7)	302 (40.3)

Father's education	university	376 (19.5)	218 (44.7)	116 (16.0)	42 (5.8)
	secondary	849 (43.9)	199 (40.8)	350 (48.3)	300 (41.7)
	vocational or lower	707 (36.6)	71 (14.5)	258 (35.6)	378 (52.5)

Mother's education	university	323 (16.5)	183 (37.3)	101 (13.6)	39 (5.4)
	secondary	1093 (55.7)	276 (56.2)	465 (62.6)	352 (48.4)
	vocational or lower	546 (27.8)	32 (6.5)	177 (23.8)	337 (46.3)

Mother's unemployment	no	1603 (81.3)	443 (89.5)	632 (88.4)	575 (81.0)
	yes	368 (18.7)	52 (10.5)	83 (11.6)	135 (19.0)

Father's unemployment	no	1653 (87.0)	446 (93.9)	615 (83.1)	545 (74.0)
	yes	247 (13.0)	29 (6.1)	125 (16.9)	191 (26.0)

Doubts about affordability of further study	never	994 (50.3)	224 (45.3)	346 (46.8)	424 (57.1)
	rarely	411 (20.8)	127 (25.7)	160 (21.6)	124 (16.7)
	sometimes	339 (17.1)	81 (16.4)	138 (18.6)	120 (16.2)
	very often, often	234 (11.8)	63 (12.7)	96 (13.0)	75 (10.1)

Perceived social support *	from mother	9.77 (3.36)	9.95 (3.44)	9.62 (3.32)	9.81 (3.34)
	
	from father	11.74 (4.29)	11.67 (4.27)	11.52 (4.14)	12.00 (4.45)
	
	from friends	9.84 (2.84)	9.86 (2.84)	9.81 (2.82)	9.87 (2.84)

School atmosphere	friendly	969 (49.0)	291 (59.0)	385 (51.9)	293 (39.4)
	Bearable, bad	1009 (51.0)	202 (41.0)	357 (48.1)	450 (60.6)

Study conditions at school	excellent	512 (25.9)	127 (25.7)	165 (22.2)	220 (29.7)
	
	fairly good	1299 (65.7)	324 (65.5)	512 (68.9)	463 (62.6)
	bad	167 (8.4)	44 (8.9)	66 (8.9)	57 (7.7)

Do you like visiting school	yes	408 (20.6)	104 (21.1)	140 (18.8)	164 (22.0)
	yes, a bit	968 (48.8)	240 (48.6)	354 (47.6)	374 (50.2)
	no	606 (30.6)	150 (30.4)	249 (33.5)	207 (27.8)

Sense of coherence *	manageability	13.48 (2.95)	13.70 (2.73)	13.52 (2.87)	13.27 (3.15)
	
	meaningfulness	13.92 (2.97)	13.86 (2.90)	13.93 (2.94)	13.95 (3.05)
	
	comprehensibility	15.43 (3.60)	15.72 (3.41)	15.29 (3.32)	15.38 (3.99)

In rows marked by * mean (SD) are presented, for the rest N (%) are presented.

The results of logistic regression (odds ratios and confidence intervals) are shown in table 2. In secondary specialised schools, fewer males than females reported the desire to continue further in their studies. In the model based on the whole sample we found that both the father's and mother's educational level had a significant effect, but the effect of father's education remained significant only among respondents attending grammar school, and the effect of mother's education remained significant only among respondents attending specialised secondary or vocational schools. The higher the education of the parents, the more prevalent further educational aspirations among respondents were. The effect of father's unemployment was significant in the model based on the whole sample, but we did not confirm this effect in any of the models based on the separate educational tracks. This is probably due to the smaller samples from these tracks, odds ratios being of about the same size (not shown). Father's unemployment decreased the probability of putting further education into the plan. Doubts concerning the affordability of study were a significant predictor of educational aspirations, but only in the model based on the whole sample. There was a lower probability of putting further education in the plan among those who never reported doubts about the affordability of study.

**Table 2:** The association between selected factors and educational aspirations among adolescents from different educational tracks

		All respondents	Grammar schools (highest level)	Specialised sec. schools	Vocational schools (lowest level)
Age		1.11 (1.01-1.23)		1.27 (1.08-1.50)	

Gender	female	1		1	
	male	0.61 (0.50-0.76)		0.53 (0.37-0.75)	

Father's education	vocational or lower	1	1		
	secondary	1.57 (1.22-2.00)	2.09 (0.99-4.40)		
	university	3.75 (2.59-5.43)	2.99 (1.38-6.45)		

Mother's education	vocational, or lower	1		1	1
	secondary	1.66 (1.28-2.15)		2.04 (1.37-3.03)	0.88 (0.60-1.29)
	university	2.52 (1.69-3.77)		1.88 (1.07-3.30)	3.84 (1.68-8.77)

Father's unemployment	employed	1			
	unemployed	0.64 (0.46-0.89)			

Doubts about affordability of further study	very often, often	1			
	sometimes	0.79 (0.53-1.18)			
	rarely	0.84 (0.57-1.25)			
	never	0.60 (0.42-0.86)			

Perceived social support	from mother				
	
	from father	0.95 (0.93-0.98)		0.93 (0.89-0.97)	0.90 (0.89-0.99)
	
	from friends			1.08 (1.01-1.15)	

School atmosphere	bearable, bad	1			
	friendly	1.44 (1.15-1.80)			

Do you like visiting school	no	1	1	1	1
	yes, a bit	1.43 (1.12-1.84)	2.38 (1.29-4.38)	1.68 (1.15-2.44)	1.54 (0.96-2.47)
	yes	2.40 (1.73-3.23)	2.98 (1.25-7.09)	4.18 (2.48-7.05)	2.88 (1.68-4.93)

Sense of coherence	manageability	1.07 (1.03-1.11)		1.10 (1.04-1.17)	
	
	meaningfulness		1.12 (1.02-1.23)		
	
	comprehensibility				1.07 (1.02-1.12)

Logistic regression, forward stepwise conditional, OR (95% CI) are presented

A higher score indicates less social support.

The effect of social support from the father remained statistically significant among respondents attending secondary specialised and vocational school, but disappeared among respondents attending grammar school. We found a significant effect of social support from friends only among respondents attending secondary specialised schools. Those who reported study aspirations in their future were characterized by higher perceived social support from the father, but lower perceived social support from friends.

The effect of school atmosphere was significant in the model based on the whole sample, but we did not find any effect in the models based on the separate educational tracks. Those who reported a friendly school atmosphere planned further study with higher probability compared with their peers reporting bearable or bad school atmosphere. The effect of attitudes towards school remained significant in all three educational tracks. Affinity with school increased the probability of putting further education into the plan. The effect of manageability remained significant only among respondents attending specialised secondary school, and we found a significant effect of meaningfulness among respondents attending grammar school as well as a significant effect of comprehensibility among respondents attending vocational schools, though these variables were not significant in the model based on the whole sample. Respondents perceiving the world as more manageable, meaningful and comprehensible were more likely to plan further study.

We did not find any significant effect of self-rated health, mother's unemployment, social support from mother or study conditions on educational aspirations in any of the explored models.

## Discussion

This study was designed to study adolescents' educational aspirations with a particular focus on different educational tracks. A better understanding of the background of educational aspirations might help to tailor more effective intervention programs aimed at enhancing success in the educational system, and consequently increasing chances in the job market. Our findings indicate that stimulating a friendly atmosphere at school, a positive attitude towards school, encouraging fathers providing social support as well as strengthening adolescents' sense of coherence might increase the educational aspirations of young people. Adolescents in different educational tracks require different approaches. Only attitudes towards school proved to be a significant factor of educational aspiration in all three educational tracks. While educational aspiration among grammar school students seems to be sensitive to the father's education, among specialised secondary schools and vocational school students the mother's education and perceived social support from fathers contribute significantly to educational aspiration.

Based on the literature [7], we expected differences in the occurrence of educational aspirations with regard to the type of educational track, and our findings were in line with this. The higher a student's educational track level, the higher the probability that he or she will put further education into the plan. Allocation to a specific educational track is determined by several factors, including cognitive capacity, teachers' recommendations and parents' and student's educational aspirations. So it might be expected that those with higher and stronger educational aspirations are more prevalent in higher educational tracks. Nevertheless, the educational system is open and flexible with regard to further education. Taking into account the crucial role of education in the demanding job market, it might be worthwhile to stimulate educational aspirations among adolescents from all educational tracks. The aim should not be university education for everyone, but the highest possible qualifications for everyone. Moreover there are at least two studies [28,29] indicating that students from poorer socioeconomic background might be disadvantaged in their formation of educational aspirations, which might be a root cause for their underachievement. Greenhalgh et al. [29] provided a qualitative analyses focused on socioeconomic variation in application to medical schools and revealed that lower participation of able pupils from poor backgrounds seems to be associated rather with identity, motivation and cultural framing of career choices rather than with lower levels of actual knowledge.

Using survey data taken from secondary school pupils, Werfhorst and Hofstede [28] examined two explanatory mechanisms for educational inequality: (1) cultural reproduction theory explaining educational inequalities through cultural differences between classes and (2) relative risk aversion theory pointing out between-class variation in the necessity of pursuing education at branching points in order to avoid downward mobility. They concluded that the cultural reproduction theory is valid for school performance, while relative risk aversion theory is valid for educational aspiration [28].

Based on health selection theory [19], we expected a significant contribution of health into the model explaining variation in educational aspirations, but our findings here were contradictory. Self-rated health, usually a good measure of several aspects of health [22], had no significant effect on educational aspirations. We did not consider the effect of psychological health, and special attention should definitely be paid to chronically ill, disabled adolescents or adolescents with higher vulnerability to illness (frequently ill), as low well-being, frequent school absence or longstanding health limitations might significantly affect the development of educational aspirations. The role of indirect health selection via disablement or unfitness is discussed by West [30].

Our findings indicate that parents' education is important for the development of educational aspirations, which is in line with the status attainment model [3,4] and supported by the literature [7,8,31-33]. Parents with higher education have more potential to create an environment stimulating higher educational aspirations, offering the stimuli needed for the development of educational capacity, and they might express their normative expectations with regard to their children's future education. After dividing the sample according to educational tracks, differences in sensitivity to parents' education were revealed: Educational aspirations among grammar school students were stimulated by a higher father's education, but among specialised secondary school students or apprentices it was a higher mother's education which played a significant role. A greater impact of a mother's education in comparison with a father's education was also found in a study by Zuckerman [34]. We previously found [35] that only one-third of adolescents are on an educational track lower than that of their parents' education. Up to 20% of adolescents moved upward, and the remaining half of the adolescents followed an educational track similar to that of their parents [35]. While educational track may have represented intergenerational mobility in the past, educational aspiration indicates potential intergenerational mobility in the future. Of particular interest is the group which might move up, i.e. those in lower educational tracks who might move up. In this group, a higher educational level of the mother seems to be a protective factor regarding downward mobility, as it is a factor contributing to upward social mobility.

Financial issues related to education are important particularly in low socioeconomic groups with scares financial resources. Investing family income in the educational status of their kids requires specific values (cultural capital) as well as the financial means (economic capital) to do so. Financial issues concern not only the costs of the education itself, but also related costs (student housing, educational tools, etc.) and predictable losses of family income (like loss of additional income from the adolescent concerned). Value issues concern the symbolic and functional importance that parents associate with the achievement of a higher educational degree. Thinking about the future, adolescents may take into account not only their own wishes, parents' values and expectations, and their estimates of possible success or failure, but also the expected costs and losses due to the study. Those who reported doubts about the affordability of study were more likely to put further education in the plan than those who never reported these doubts often or very often. This finding is in line with cultural capital theory which explains that an individuals' ability to deal with insecurity and ambivalence is part of social class based cultural capital [36]. Children "learn" in their family that the pros and cons of higher education need to be considered when planning for the future. In line with this reasoning, our results indicate that those who put further education into their plan tend to consider also the possible financial costs and consequently perceive related economic stress. In families with low financial resources the result of this consideration is likely to turn out different from those in better financial conditions. On that, our findings suggest, that for those who had „given up" further education related financial problems are no longer an issue.

Studies of educational aspirations have focused mostly on parents' involvement and parents' support for learning [9], while our study shows that a more general measure like social support is also sufficiently sensitive to catch the differences between a more and less stimulating family environment. Our findings confirmed the effect of perceived social support from the father, but not from the mother on educational aspirations, which is a contradictory finding with regard to the literature [14,15].

Ryan [37] explored the peer group as a context for the development of adolescents' motivation and achievement in school. According to that study, adolescents tended to affiliate with other students who had academic characteristics similar to their own. Peers have an influence on adolescents' liking and enjoyment of school, but not on their beliefs about the importance of school. Particularly among non-academic educational tracks, we found an association between higher perceived social support from friends and a lower probability of putting further education into the plan. It might be hypothesised that well-settled attendees of non-academic educational tracks (those perceiving high social support from friends), an environment in which educational aspirations are much less prevalent compared with the academic educational track, might tend to affiliate with their less ambitious peers, which might keep their educational aspirations low as well.

Attitudes towards school seem to be the main condition for the development of educational aspirations across all educational tracks, and not just within upper SEP adolescents, as was found in an Australian study [17]. Our findings indicate that schools may have the potential to stimulate educational aspirations and be an important player in strategies aimed at diminishing the cumulation of socioeconomic disadvantage via the educational system.

And finally, meaningfulness among grammar school students, manageability among specialised secondary, and comprehensibility among vocational school students may contribute to the probability of their putting further education into the plan, which partially supports the hypothesis of Lundberg [12] on the role of sense of coherence in shaping socioeconomic inequalities in health via the educational system. Our findings may be interpreted as meaning that social sources (supportive family and school environment) and individual sources (sense of coherence) counteract the effect of low SEP on educational aspiration.

The important strengths of our study are its high response rate and the fact that it included a wide range of variables, including adolescents' economic background (family SEP), social background (family social support, school, friends) and individual variables (gender, health, sense of coherence) in one analysis. However, we did not measure data from parents, such as their former involvement in school, and parents' previous educational aspirations. Similarly, we did not check the educational status and occupation of parents. However, the latter issue was explored by West, Sweeting & Speed [38,38], who confirmed rather good reliability of data provided by adolescent respondents upon parents' SEP. The sample selection procedure, the size of the sample and the high response rate support the representativeness of our findings regarding the adolescent population of East Slovak.

Finally, it may be questioned whether high educational aspirations are appropriate for every student. It may be that for some students, continuing their studyies in future is not a good option given their cognitive skills and other factors. This topic requires further study on the mediating effects of factors such as intelligence and learning skills [10,39] to further tailor interventions to counteract socioeconomic health differences.

## Conclusions

It is generally accepted that cultural capital forms a gateway to a better socioeconomic position for the individual, as well as to the general prosperity of society. Our findings indicate that those who need education the most expect it the least (vocational educational track). Moreover, both school and the family have the potential to stimulate educational aspirations across all educational tracks. The attitude towards school and social support from the father are the most consistent predictors of educational aspirations across all three types of education. The role of SEP is confirmed only partially. This offers important clues towards reducing socioeconomic differences.

## Competing interests

The authors declare that they have no competing interests.

## Authors' contributions

AMG and JPvD contribute to the acquisition of data. AMG, PT, TA, JPvD, and SAR contributed to the analysis and interpretation of data and were involved in drafting, revising and final approval of the manuscript.

## Pre-publication history

The pre-publication history for this paper can be accessed here:

http://www.biomedcentral.com/1471-2458/10/154/prepub
